# GW627368X inhibits proliferation and induces apoptosis in cervical cancer by interfering with EP4/EGFR interactive signaling

**DOI:** 10.1038/cddis.2016.61

**Published:** 2016-03-24

**Authors:** S Parida, I Pal, A Parekh, B Thakur, R Bharti, S Das, M Mandal

**Affiliations:** 1 School of Medical Science and Technology, Indian Institute of Technology, Kharagpur, West Bengal 721302, India; 2 National Institute of Cholera and Enteric Diseases, P-33, C.I.T. Road, Scheme XM, Beleghata, Kolkata, West Bengal 700010, India

## Abstract

PGE2, the major product of cyclooxygenases implicated in carcinogenesis, is significantly upregulated in cervical cancer. PGE2 *via* prostanoid receptor EP4 stimulates proliferation and motility while inhibiting apoptosis and immune surveillance. It promotes angiogenesis by stimulating the production of pro-angiogenic factors. The present study demonstrates GW627368X, a highly selective competitive EP4 antagonist, which hinders cervical cancer progression by inhibiting EP4/epithelial growth factor receptor (EGFR) interactive signaling. GW627368X reduced protein kinase A (PKA) phosphorylation which in turn leads to decreased cAMP response element-binding protein (CREB) activation. Decreased PKA phosphorylation also directly enhanced Bax activity and in part reduced glycogen synthase kinase 3 (GSK3)*β* phosphorylation. Owing to the interactive signaling between EP4 and EGFR, GW627368X lowered EGFR phosphorylation in turn reducing Akt, mitogen-activated protein kinase (MAPK) and GSK3*β* activity significantly. Sublethal dose of GW627368X was found to reduce the nuclear translocation of *β*-catenin in a time dependent manner along with time-dependent decrease in cytoplasmic as well as whole-cell *β*-catenin. Decreased CREB and *β*-catenin transcriptional activity restricts the aberrant transcription of key genes like EP4, cyclooxygenase (COX)-2, vascular endothelial growth factor and c-myc, which ultimately control cell survival, proliferation and angiogenesis. Reduced activity of EGFR resulted in enhanced expression of 15-hydroxyprostaglandin dehydrogenase increasing PGE2 degradation thereby blocking a positive feedback loop. In xenograft model, dose-dependent decrease in cancer proliferation was observed characterized by reduction in tumor mass and volume and a marked decrease in Ki67 expression. A diminished CD31 specific staining signified decreased tumor angiogenesis. Reduced expression of pAkt, pMAPK, pEGFR and COX-2 validated *in vitro* results. GW627368X therefore effectively inhibits tumor survival, motility, proliferation and angiogenesis by blocking EP4/EGFR interactive signaling. EP4 is a potent therapeutic target in cervical cancer and can be explored in combination with conventional therapies to attain superior outcomes and to overcome complications associated with organ toxicities, therapeutic resistance and disease relapse.

Cervical cancer is the second most common cancer affecting women worldwide and second most common killer of women in developing nations accounting 86% of cervical cancer deaths. In India, 122 844 women are diagnosed with cervical cancer annually and 67 477 die from the disease.^1^ In US, 12 900 cases and 4100 deaths are expected in 2015. Despite a declining trend, incidence and mortality has normalized in the past decade even in developed nations.^2^ Despite recent advances in chemo and radiotherapy, treatment failure and disease relapse remain to be major problems. Currently available prophylactic vaccines against a subset of HR-HPVs hold promise for reducing burden in future generations. However, these vaccines do not protect women already inflicted with the infection. Efforts for developing therapeutic vaccines against viral antigens E6 and E7 are underway and despite being able to induce viral antigen specific cytotoxic T cells in human, they have proven largely ineffective in treating HPV-induced cervical cancer.^3^ The vaccines are yet to be approved in many countries and mass vaccination in developing countries is difficult due to lack of infrastructure and other socioeconomic factors. Development of newer and more effective therapeutic agents therefore remains indispensable.

COX-2 upregulation and enhanced PGE2 synthesis in cervical carcinoma is well known and it regulates neoplastic cell function *via* the EP2 and EP4 receptors.^4^ COX-2 expression is correlated with higher vascular endothelial growth factor (VEGF) expression, lymphangiogenesis and lymph node metastasis in cervical cancer.^5^ Most of the effects of COX-2 are attributed to its enzymatic product PGE2.^6^ However, another isoform of the enzyme, COX-1, earlier considered to have housekeeping roles, is found to be overexpressed in cervical cancer consistent with COX-2 and PGE2.^7^ Studies suggest that COX-1 upregulation modulates the expression of COX-2, PGE2 receptors and angiogenic factors in cervical cancer.^7^ PGE2 stimulates cell proliferation and motility while hindering immune surveillance and preventing apoptosis.^8^ PGE2 stimulates angiogenesis by stimulating the synthesis of pro-anigiogenic factors including VEGF. Stimulation of EP4 by PGE2 also activates TCF-*β*-catenin-mediated transcription that in turn leads to increased expression of variety of genes implicated in cancer-like cyclin D1 and c-myc.^8^ Moreover, PGE2 has distinct organ specific effects as it is associated with estrogen biosynthesis. Estrogen biosynthesis is catalysed by aromatase, product of *CYP19* gene. Binding of PGE2 to EP4 receptor stimulates adenylyl cyclase activity enhancing cyclic adenosine monophosphate production which induces the transcription of gene encoding aromatase via CREB.^8^ Consequently there is increased biosynthesis of aromatase enzyme. It is important mediator in cervical carcinogenesis as the uterine cervix is highly responsive to estrogen. Recently it has been reported that estrogen and its nuclear receptors promote cervical cancer in combination with HPV.^3^ Estrogen is also known to be independently capable of inducing cervical epithelial hyperplasia.^3^

Folds (106) higher expression of EP4 is reported in cervical cancer compared with normal controls.^4^ Moreover, EP4, unlike other prostanoid receptors, associates with multiple signaling pathways involved in carcinogenesis.^9^ In the present study we demonstrate EP4 prostanoid receptor as a potential therapeutic target in cervical cancer using GW627368X, a highly selective, competitive EP4 antagonist.^10^ We explored the interactive signaling between EP4 prostanoid receptor and the epidermal growth factor receptor (EGFR). GPCRs and EGFR are known to be simultaneously overexpressed and contribute to aberrant proliferation of cancer by activating autocrine pathways.^11^ PGE2 can stimulate EGFR in many complex and context specific mechanisms.^11^ PGE2, via the EP4 receptor, stimulates cyclic AMP-protein kinase A (PKA) pathway which increases expression of EGFR ligand amphiregulin.^8^ EGFR transregulation by PGE2 is also known to be mediated by intracellular Src-dependent mechanism independent of extracellular EGFR ligands.^8^ PGE2 stimulates matrix metalloproteinase activity resulting in the shedding of active EGFR ligands from the plasma membrane, which in turn magnifies EGFR signaling and enhances DNA synthesis in certain cancers.^11^ On the other hand, activation of EGFR signaling leads to increase in MAPK activity resulting in activator protein-1-mediated induction of COX-2 transcription and enhanced synthesis of PGE2.^8, 11, 12^ Stimulation of EGFR signaling also inhibits expression of 15-hydroxyprostaglandin dehydrogenase, the key enzyme for catabolism of PGE2, leading to enhanced accumulation of PGE2 in the tumor vicinity due to increased production and decreased degradation.

## Results

### GW627368X inhibits the proliferation of cervical cancer cells in time and dose-dependent manner by inducing apoptosis

**Figure 1:**
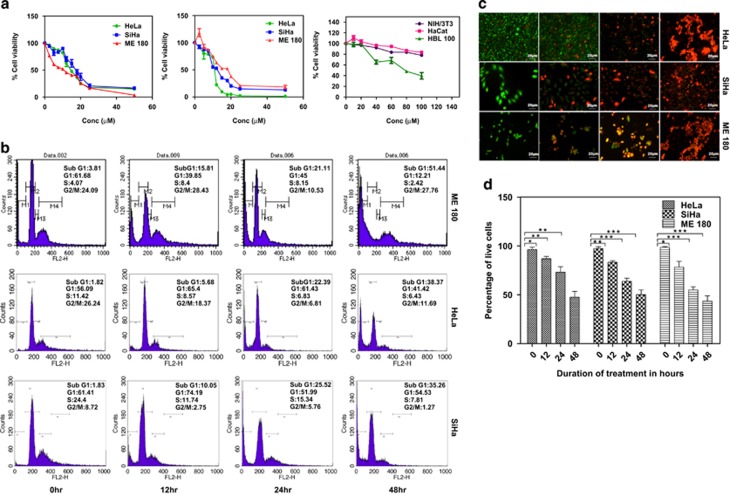
Anti-proliferative potential of GW627368X. (**a**) Dose-dependent inhibitory effect of GW627368X on growth of cervical cancer cell lines. Cervical cancer cell lines HeLa, SiHa, ME 180 and normal cell lines NIH/3T3, HaCat and HBL-100 were treated with various concentrations of GW627368X for 24 and 48 h. Points, average±S.D., representative of three independent experiments each performed in triplicates, *P*<0.05. (**b**) Cell cycle analysis. Indicated cell lines were treated slightly lower than IC_50_ (48 h) dose of the drug (9, 10 and 10 *μ*M for Hela, SiHa and ME 180, respectively) in treatment groups and 0.1% DMSO in control for indicated time periods. (**c** and **d**) Live dead assay. (**c**) Micrographs representing cells stained with Calcein AM and Ethidium bromide homodimer after treating the cells for indicated time periods. Viable cell population appears green (calcein AM) whereas non-viable/dead cells appear as orange/red (ethidium bromide) in the fluorescent micrographs. (**d**) Graphical representation of percentages of live and dead cells represented as histogram, Values presented as mean±S.D.; *P<0.05, **P<0.01, ***P<0.001

**Figure 2:**
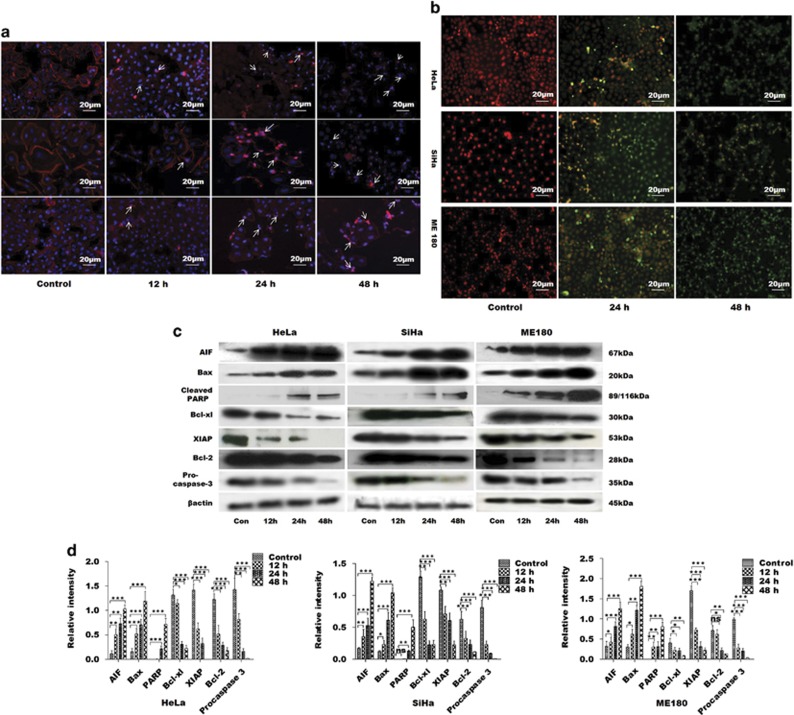
GW627368X induces apoptosis in cervical cancer cells HeLa, SiHa and ME 180 in time-dependent manner. Cells treated with 9, 10 and 10 *μ*M GW627368X for Hela, SiHa and ME 180, respectively, for indicated time periods, (**a**) stained with Rhodamine phalladoin and counter stained with DAPI and images captured at × 20 magnification. Nuclear fragmentation and disruption of actin cytoskeleton, characteristic of apoptotic cell death, was observed (**b**) TUNEL assay was performed and fluorescent images were captured. Time-dependent increase in TUNEL-positive green nuclei indicated induction of apoptosis, (**c** and **d**) Western blot analysis of apoptotic proteins, (**c**) Blots depicting expression profile of apoptotic and anti-apoptotic proteins in cervical cancer cells treated with GW627368X for indicated time periods compared with control treated with 0.1% DMSO, *β*-actin used as loading control (**d**) Quantification of protein expression by densitometry, normalized to *β*-actin, each representative of three different experiments each performed in triplicates, values presented as mean±S.D.; *P<0.05, **P<0.01, ***P<0.001

The potential of GW627368X to inhibit proliferation of cervical cancer cell lines was evaluated by 3-(4,5-dimethylthiazol-2-yl)-2,5 diphenyltetrazolium bromide (MTT) assay. The IC_50_ values for HeLa, SiHa and ME 180 cell lines were found to be 17.44±0.88, 29.92±0.83 and 23.22±0.95 *μ*M, respectively, at 24 h and 9.082±0.8, 11.3±0.91 and 11.16±0.94 *μ*M, respectively, at 48 h (Figure 1a). A similar time-dependent decrease in cell viability was also observed from live/dead assay (47.39±5.992, 50.06±4.894 and 43.45±5.417% in HeLa, SiHa and ME 180, respectively, after 48 h; Figures 1c and d). Minimal cytotoxicity was observed in case of normal cells evident from IC_50_, 110.5±0.84 *μ*M, 488.2±0.8 *μ*M and 171.9±0.872 *μ*M for HaCat, NIH/3T3 and HBL-100, respectively (Figure 1a). To further study the chronology of cellular events after GW627368X treatment, cell cycle analysis was performed by flow cytometry with treatment slightly lower than IC_50_ value (9, 10 and 10* μ*M for HeLa, SiHa and ME 180, respectively). A time-dependent accumulation in sub G0/G1 phase was observed. Percentage of sub G0/G1 population substantially increased to 45.46±2.1, 41.39±3.7 and 57.61±1.3 when treated compared with 1.82±0.9, 1.83±0.7 and 3.81±0.3 in control in HeLa, SiHa and ME 180, respectively (Figure 1b). GW627368X inhibits growth in cervical cancer cells by inducing apoptosis was indicated by morphological studies using DAPI/Rhodamine phalloidin staining (Figure 2a). Typical changes in cell morphology including cell shrinkage, disruption of actin filaments, nuclear fragmentation and apoptososme formation was observed. Apoptotic cell death was further confirmed by a time-dependent increase in terminal deoxynucleotidyl transferase dUTP nick-end labeling (TUNEL)-positive green nuclei (Figure 2b). Activation of pro-caspase 3 is important for induction of apoptosis further leading to increased PARP cleavage. A time-dependent decrease in cellular levels of pro-caspase 3 and increase in levels of cleaved PARP was observed in all cell lines (Figures 2c and d). The PGE2/EP4 pathway is known to suppress apoptosis in cervical cancer cells by preventing the translocation of pro-apoptotic protein BAX into the mitochondria. Time-dependent expression levels of pro-apoptotic proteins BAX and apoptosis-inducing factor were studied following drug treatment by western blot analysis and showed a significant increasing pattern. Further a decrease in levels of anti-apoptotic proteins, Bcl-2, Bcl-xl and XIAP was observed in a time-dependent manner in all the cell lines (Figures 2c and d). These findings could be correlated with the results of MTT as well as TUNEL and Live/Dead assays.

### GW627368X impedes migration and invasion of cervical cancer cells

**Figure 3:**
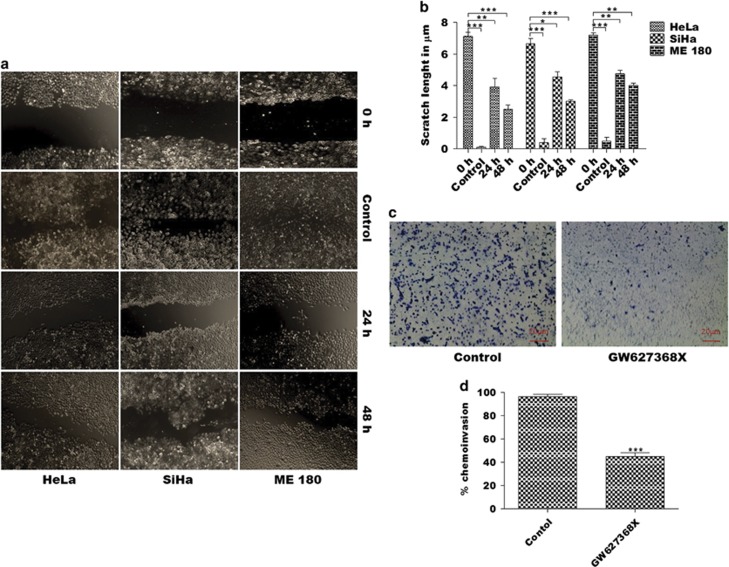
GW627368X impedes migration and invasion of cervical cancer cells. (**a** and **b**) Wound healing assay. Cells were allowed to grow in monolayers in six-well plates and the monolayer was wounded with a pipette tip. Cells were then treated with one third IC_50_ of GW627368X. The control cells were treated with 0.1% DMSO. (**a**) The wounds were observed and photographed till 48 h at regular intervals. (**b**) Graphical representation of wound size at different time points. (**c** and **d**) Boyden chamber assay. (**c**) Micrographs representing invasion of HeLa, SiHa and ME 180 cells following treatment with GW627368X. (**d**) Graph depicting percentage of cells invading the inserts of Boyden chamber coated with matrigel, representative of three different experiments each performed in triplicates, values presented as mean±S.D.; *P<0.05, **P<0.01, ***P<0.001

Potential of GW627368X to impede migration and invasion of cervical cancer cells was determined from wound healing and Boyden chamber assays. Considerable wound closure was observed in control cells after 48 h (2.5±0.2887, 3.033±0.0819 and 4.033±0.1453 *μ*m, *N*=3 in HeLa, SiHa and ME 180, respectively). However, little healing was observed in drug treated groups (7.140±0.2358, 6.667±0.3283 and 7.2±0.15281453 *μ*m, *N*=3 in HeLa, SiHa and ME 180, respectively; Figures 3a and b). Boyden chamber assay revealed about 30% reduction (*P*=0.0002) in invasive potential of ME 180 cells, most invasive of all the cell lines (Figures 3c and d).

### GW627368X inhibits angiogenesis, an important phenomenon in carcinogenesis

**Figure 4:**
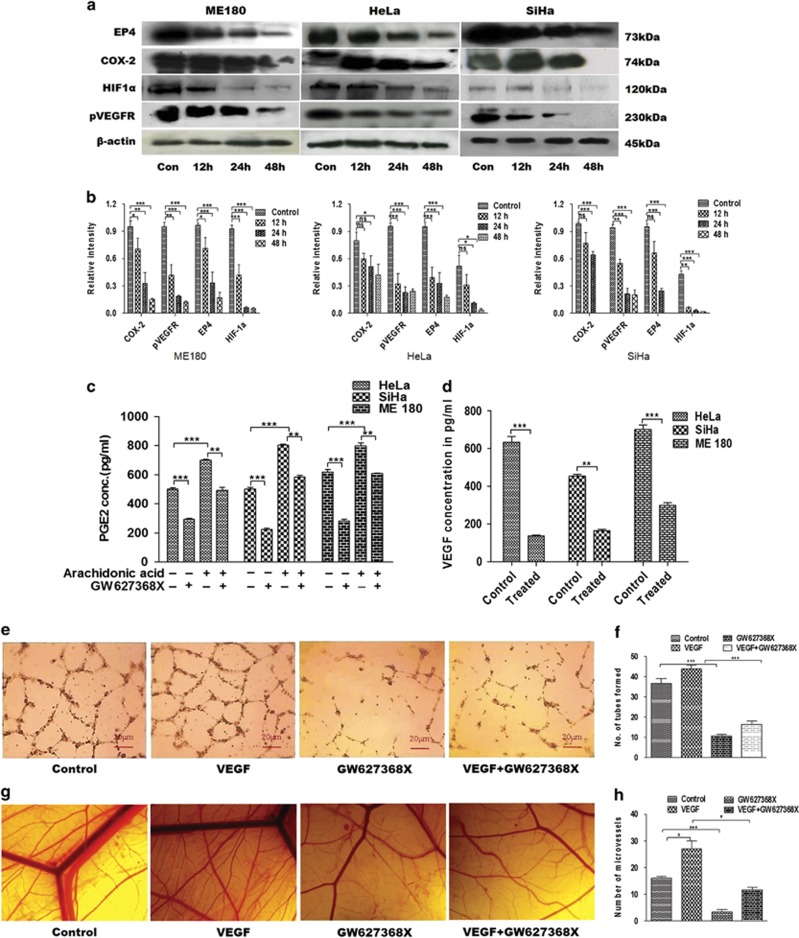
Anti-angiogenic potential of GW627368X. Cyclo-oxygenase/PGE2 axis is known to have prominent role in angiogenesis. (**a**) Western blot analysis of major players of COX-PGE2 axis in cervical cancer cell lines treated with GW627368X (9, 10 and 10* μ*M for Hela, SiHa and ME 180, respectively) for different time points and control treated with 0.1% DMSO. (**b**) Quantification of protein expression by densitometry, normalized against *β*-actin, each representative of three different experiments performed in triplicates, values presented as mean±S.D.; *P<0.05, **P<0.01, ***P<0.001. (**c**) Quantitative analysis of the PGE2 concentration in conditioned media of GW627368X treated cells (9, 10 and 10 *μ*M for Hela, SiHa and ME 180, respectively), stimulated or unstimulated by 10 *μ*M arachidonic acid by ELISA. Graphs representative of three different experiments each performed in triplicates, values presented as mean±S.D.; *P*<0.05, **P<0.01, ***P<0.001. (**d**) Quantitative estimation of the secretory VEGF concentration in conditioned media of GW627368X treated cells (9, 10 and 10* μ*M for Hela, SiHa and ME 180 respectively) by ELISA. Graphs representative of three different experiments each performed in triplicates, values mean±S.D.; *P*<0.05, **P<0.01, ***P<0.001. (**e** and **f**) *In vitro* tube formation assay. (**e**) HUVECs were seeded (7.5 × 10^3^ cells/well) into a 96-well tissue culture plate coated with 50 *μ*l matrigel; VEGF (25 ng/ml), GW627368X (IC_50_ for 48 h/3)or both were added and incubated in HUVEC growth medium in 37 °C, 5% CO_2_ incubator. Tube formation was observed for 24 h and images were taken at × 10 magnification (**f**) Number of capillary-like structure formed by HUVECs in each condition was counted under light microscope after 24 h in four independent experiments. Data presented as mean±S.D., *P*<0.05, **P<0.01, ***P<0.001. (**g** and **h**) Chorioallantoic membrane (CAM) assays. (**g**) Photomicrographs representing developing chick embryos implanted with sterile filter disks loaded with serum-free media as control and serum-free media supplemented with VEGF (25 ng/ml), GW627368X (IC_50_ for 48 h/3) or both to observe the angiogenic response (**h**) The number of blood vessels formed in each test group was counted under stereo microscope and plotted to evaluate the extent of angiogenic response. Data presented as mean±S.D., *P*<0.05, **P<0.01, ***P<0.001 of three independent experiments performed in triplicates

Angiogenesis, an indispensable phenomenon in tumor progression, is regulated by numerous angiogenic factors; the most important one being VEGF. Numerous studies have indicated that the role of prostaglandin E2 (PGE2) and VEGF are closely intertwined in regulation of tumor angiogenesis. PGE2 has been reported to promote angiogenesis independently as well as by stimulating the production of VEGF. In addition, reports also suggest that effect of VEGF on angiogenesis, at least in part, is mediated by PEG2 synthesis. An increased PGE2 signaling via EP4 receptor is known to activate a positive feedback loop thus enhancing COX-2, PGE2, EP4 and VEGF expression further amplifying angiogenic response.^5^ Therefore, GW627368X, an EP4 receptor inhibitor, was hypothesized to have anti-angiogenic potential. To determine anti-angiogenic properties of GW627368X, levels of PGE2 and VEGF in conditioned medium was quantified by enzyme-linked immunosorbent assay (ELISA). Significant decrease in levels of PGE2 on treatment was observed in all cell lines. PGE2 levels significantly rose on stimulation with 10 *μ*M arachidonic acid (700.4±6.104, 802.9±8.395, 798.6±19.01 pg/ml,*N*=3) compared with control (500.8±9.616, 500.6±12.07, 618.0±18.51 pg/ml, *N*=3) in HeLa, SiHa and ME 180, respectively (Figure 4c). However, comparable decrease in PGE2 levels in conditioned medium was observed in both stimulated (493.4±20.31, 583.5±12.58, 608.1±2.915 pg/ml, *N*=3 for HeLa, SiHa and ME 180, respectively) and unstimulated cells (294.2±6.807, 225.5±7.945, 281.4±12.13 pg/ml, *N*=3 for HeLa, SiHa and ME 180, respectively; Figure 4b). Consequently, decrease in secretory VEGF was observed in all cell lines when treated with GW627368X (135.5±4.16, 163.5±9.034, 300.0±11.36 pg/ml, *N*=3 for Hela, SiHa, ME 180, respectively) compared with untreated control (634.6±29.92, 454.4±8.695, 701.4±24.12, *N*=3 Hela, SiHa, ME 180, respectively; Figure 4d). Further, expression profiles of COX-2, EP4, p-vascular endothelial growth factor receptor and HIF1*α* at different time periods of drug treatment was inspected by western blot analysis. Time-dependent decrease in expression level was observed in each protein (Figures 4a and b). Anti-angiogenic potential of GW627368X was analyzed *in vitro* by tube formation assay. The number of tube like capillaries formed by HUVECs significantly constrained when treated with the drug (Figures 4e and f). These *in vitro* results were further validated *in vivo* by chorioallantoic membrane assay. VEGF treatment increased number of small blood vessels in developing chick embryo compared with control, however, an avascular zone was observed around the area of drug treatment in the third group (Figures 4g and h). The number of capillaries were counted and plotted.

### GW627368X inhibits proliferation and induces apoptosis in cervical cancer cells via multiple pathways involving EP4/PKA/CREB, EGFR/Akt/GSK3*β*/*β*-catenin and EGFR/Ras/MAPK/CREB

**Figure 5:**
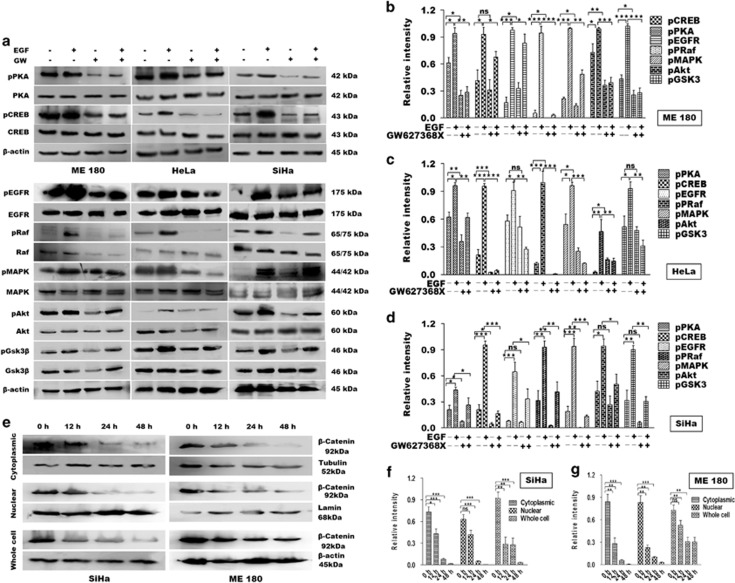
GW627368X interferes with EP4/EGFR interactive signaling in cervical cancer. (**a**–**d**) Cervical cancer cells were treated with 9, 10 and 10 *μ*M of drug for HeLa, SiHa and ME 180, respectively, followed by stimulation with 25 ng/ml of recombinant EGF for 30 min and equal amount of proteins (50 *μ*g) were analyzed by western blotting. (**a**) Representative blots showing western blot analysis of key proteins. (**b–d**) Relative levels of phosphorylation of each protein was determined by densitometry. Data presented as mean±S.D., *P*<0.05, **P<0.01, ***P<0.001 of three independent experiments performed in triplicates. (**e**) Cells were treated with one third of IC_50_(48 h) of the drug, incubated for different time periods and protein was extracted from whole cell, nuclear and cytoplasmic fractions and relative *β*-catenin expression in equal amount of protein (50 *μ*g) was analyzed by western blot. (**f** and **g**) The relative levels of protein expression was determined by densitometry. Data presented as mean±S.D., *P*<0.05, **P<0.01, ***P<0.001 of three independent experiments performed in triplicates

EP4 receptors blockade with GW627368X resulted in significant reduction in proliferative potential and induced time and dose-dependent apoptosis in cervical cancer cells. To trace the cellular pathways affected by GW627368X, cells were stimulated with EGF and phosphorylation status of downstream effectors was studied by western blot. EP4 has been previously reported to be coupled with PKA or adenylyl cyclase, thus elevating intracellular cyclic AMP levels ultimately leading to increased phosphorylation of CREB transcription factor which further augments the transcription of multiple genes.^13^ Interestingly, in our study along with decreased PKA and CREB phosphorylation, we observed a significant downregulation of EGFR phosphorylation (Figures 5a–d). Further, on western blot analysis we observed a time-dependent decrease in *β*-catenin expression with respect to control cells (Figures 5e and g). These findings opened up the possibility of cross talk between EP4/CREB and EP4/EGFR pathways. On detailed analysis we found that treatment with GW627368X significantly reduced phosphorylation levels of EGFR, Ras, Akt, GSK3*β* and MAPK in both stimulated and unstimulated cells while levels of total proteins remained unchanged (Figures 5a–d). On treating the cells with a sub-lethal dose of the drug (one-third of IC_50_), a time-dependent decrease in expression of *β*-catenin in SiHa and ME 180 cells along with decreased nuclear accumulation was found (Figures 5e–g). Our results thus indicated that GW627368X hindered proliferation and induced apoptosis in cervical cancer cells via the cyclic adenosine monophosphate/PKA/CREB as well as pathway involving EGFR/Ras/MAPK/CRB and Akt/GSK3*β*/ *β*-catenin. Reduced PKA phosphorylation also stimulated Bax-induced apoptosis. Decreased activity of CREB and *β*-catenin can be implicated in reduced levels of VEGF, COX-2 and PGE2.

**Figure 6:**
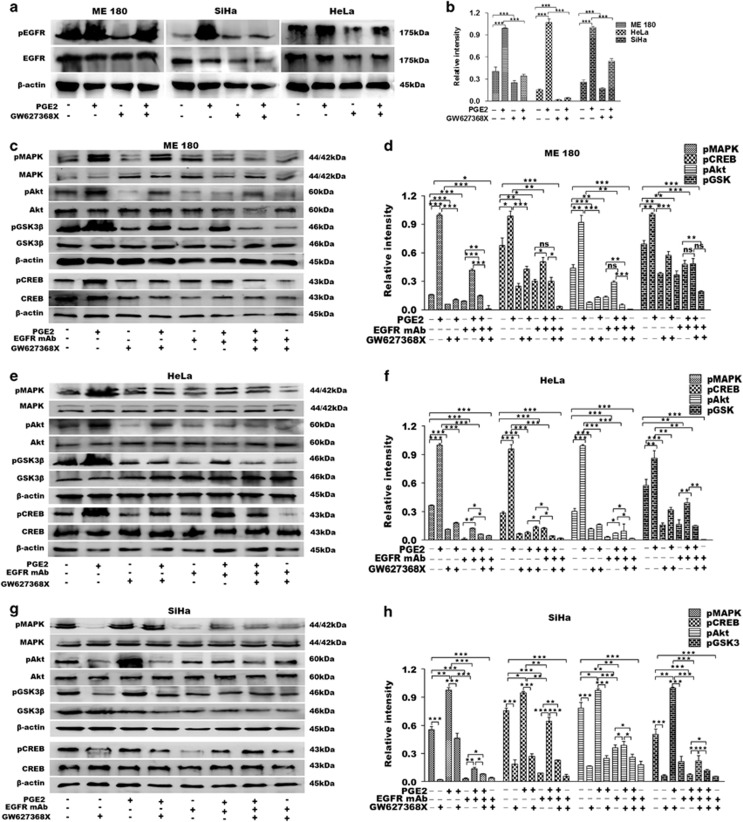
Hindrance of EP4/EGFR cross-talk validated by exogenous overexpression of PGE2 and EGFR blockade. (**a** and **b**) Cells were stimulated with exogenous PGE2 (100 nM) and phosphorylation status of stimulated or unstimulated cells with or without drug treatment was studied by western blot. (**a**) Representative blots showing phosphorylation levels of EGFR in cervical cancer cell lines. (**b**) The relative levels of pEGFR expression was determined by densitometry. Data presented as mean±S.D., *P*<0.05, **P<0.01, ***P<0.001 of three independent experiments performed in triplicates. (**c**–**h**) Cells were stimulated with exogenous PGE2 (100 nM) and EP4, EGFR or both were blocked with GW627368X9 (9, 10 and 10 *μ*M of drug for HeLa, SiHa and ME 180), EGFR mAb (20 ng/ml) or both and phosphorylation status of key downstream molecules were studied by western blot. (**c**, **e** and **g**) Representative blots showing activation levels of key regulator proteins in ME 180, HeLa and SiHa, respectively. (**d**, **f** and **h**) The relative levels of protein expression was determined by densitometry. Data presented as mean±S.D., *P*<0.05, **P<0.01, ***P<0.001 of three independent experiments performed in triplicates

**Figure 7:**
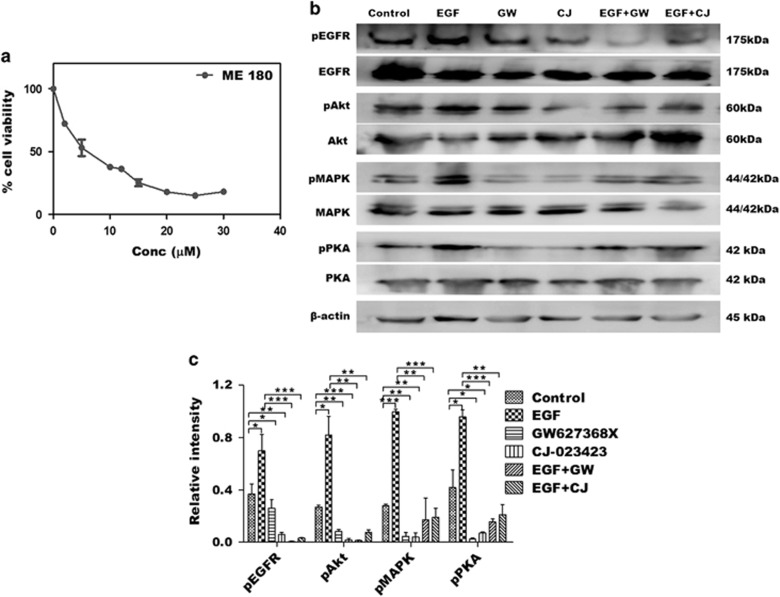
Validation of the effect of EP4 inhibition in cervical cancer using CJ-023423, a highly selective EP4 inhibitor as positive control. (**a**) Dose-dependent inhibitory effect of CJ-023423 on growth of cervical cancer cell line, ME 180. ME 180 cells were treated with various concentrations of GW627368X for 48 h. Points, average±S.D., representative of three independent experiments each performed in triplicates, *P*< 0.05. (**b** and **c**) ME 180 cells were treated with GW627368X (10 *μ*M) and CJ-023423(7 *μ*M) followed by stimulation with EGF. The relative phosphorylation levels of important signaling mediators were studied by western blot analysis. (**b**) Representative blots showing western blot analysis of key proteins. (**c**) The relative levels of phosphorylation of each protein was determined by densitometry. Data presented as mean±S.D., *P*<0.05, **P<0.01, ***P<0.001 of three independent experiments performed in triplicates

To further validate our hypothesis that GW627368X hinders cervical carcinogenesis *via* EP4/EGFR interactive signaling, we studied the phosphorylation status of key effector molecules after exogenously over-expressing PGE2. On stimulating the cells with PGE2, a highly significant amplification of pEGFR was observed while the total EGFR levels remained unaffected (Figures 6a and b). It markedly reduced post GW627368X treatment in both stimulated and unstimulated cells (Figures 6a and b). An increase in ambient PGE2 resulted in increased CREB phosphorylation as well as increase in pMAPK, pAkt and pGSK3*β* (Figures 6c–h). GW627368X being a competitive EP4 antagonist prevented binding of PGE2 to its receptor bringing down the levels pMAPK, pAkt, pGSK3*β* and pCREB (Figures 6c–h). Interestingly, we observed a similar downregulation of pAkt, pGSK3*β*, pMAPK and relatively lower, but significant downregulation of pCREB on blockade of EGFR using mAb without GW627368X (Figures 6c–h). Activation of these effectors was further reduced on simultaneous blockade of both EP4 and EGFR (Figures 6c–h) suggesting a crosstalk of pathways being affected. The results obtained above were validated by comparing it with another highly selective EP4 receptor inhibitor, CJ-023423 (Figure 7). The IC_50_ of CJ-023423 against ME 180 was determined to be 7.611±0.9416 *μ*M for 48 h (Figure 7a). Phosphorylation studies were then performed treating ME 180 cells with either 7 *μ*M CJ-023423 or 10 *μ*M GW627368X (Figure 7b) and the results compared (Figure 7c). A similar and slightly higher inhibition of EGFR, Akt, PKA and MAPK phosphorylation was observed, validating our previous results.

### GW627368X hinders tumor progression *in vivo*

**Figure 8:**
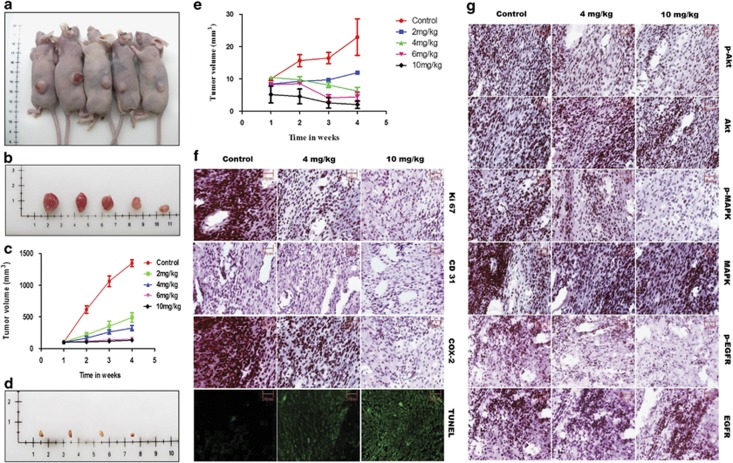
GW627368X hinders tumor progression *in vivo.* Subcutaneous tumor-bearing nude mice were treated with GW627368X orally ranging from 0 to 10 mg/Kg body weight, thrice a week, for 4 weeks. (**a**) Representative picture showing subcutaneous tumor (ME 180)-bearing nude mice of different treatment groups. (**b**) Representative picture showing excised tumors from corresponding treatment groups of ME 180 xenografts. (**c**) Graph representing change in tumor volume (group average; ME 180 xenografts) in each test group measured every week. Data presented as mean±S.D. (*n*=5), *P*=0.0125. (**d**) Representative picture showing tumors excised from different treatment groups of SiHa xenografts. (**e**) Graph representing change in tumor volume (group average; SiHa xenografts) in each test group measured every week. Data presented as mean±S.D. (*n*=5), *P*=0.0002. Paraffin-embedded excised tumors from different treatment groups of ME 180 xenografts were sectioned, processed and (**f**) *In situ* TUNEL assay to assess induction of apoptosis within tumor and IHC specific for COX-2, Ki67(proliferation marker) and CD31(anigiogenesis marker) were performed (**g**) Photomicrographs representing IHC performed for specific signaling proteins, pMAPK, MAPK, pAkt, Akt, pEGFR and EGFR

The effect of GW627368X on ME 180 and SiHa xenograft models were studied by treating subcutaneous tumor-bearing nude mice with oral dosage of the drug ranging from 0 to 10 mg/Kg body weight, thrice a week, for 4 weeks. Significant reduction in volume of tumors with increase in drug dose was observed in both cases (*P*=0.0125, *N*=5 for ME 180 and *P*=0.0002, *N*=5 for SiHa; Figure 8a). In ME 180 xenograft, final tumor volume in highest treatment group reduced to 133.7±17.28 mm^3^ compared with 1348±53.63 mm^3^ in control group (*P*<0.0001, *N*=5; Figures 8b and c). Cervical cancer cell line SiHa is known to form poorly differentiated tumor in nude mice. Though a very similar regression in tumor was observed, clear results could not be obtained in case of SiHa xenograft because of very low initial tumor volume. Final tumor volume in highest treatment group reduced to 2.033±1.184 mm^3^ compared with 22.92±5.641 mm^3^ in control group (*P*=0.023, *N*=5; Figures 8d and e). The weight of the animals remained grossly similar from beginning to end of treatment. All further staining and immunohistochemistry (IHC) based studies were performed on tissue sections obtained from ME 180 xenografts. Inhibition of proliferation was evident from IHC specific to Ki67 indicating significantly lower expression in treatment groups compared with control (Figure 8f). DNA fragmentation within tumor tissue due to apoptosis promoting effect of GW627368X was evident from TUNEL assay (Figure 8f). Decrease in angiogenesis *in vivo* was also evident from decreased intensity of CD31 staining in treatment groups *versus* controls (Figure 8f). Expression profiles of important components of signaling pathway by IHC. A decreased expression of COX-2, pAkt, pMAPK and pEGFR was observed in treatment groups whereas that of tAkt, tMAPK and tEGFR differed slightly or remained unchanged (Figure 8g). These results *in vivo* were consistent with results obtained *in vitro*.

## Discussion

In the present study, we demonstrated the potential therapeutic efficacy of GW627368X in inhibiting cervical carcinogenesis *in vitro* and *in vivo* by hampering EP4-mediated multiple signaling cascades. GW627368X is a potent, highly selective, competitive EP4 prostanoid receptor antagonist with Ki value of 100 nM.^10^ EP4 is known to be significantly upregulated in cervical cancer consistent with overexpression of COX-1, COX-2 and their product, PGE2.^7, 14, 15, 16, 17^ It has also been associated with poor chemotherapeutic outcome and lymph node metastasis in cervical cancer.^5, 7, 18, 19^ In our study, GW627368X was used as a selective blocker in three HPV-positive cervical cancer cell lines, HeLa, SiHa and ME 180, and was found to diminish their viability in a time- and dose-dependent manner by inducing apoptosis evident from morphological studies and TUNEL assay. Protein profiling by western blot revealed an elevated BAX to Bcl-2 ratio which leads to apoptosis by caspase-3 activation and PARP cleavage. In addition, a prominent increase in apoptosis-inducing factor and decrease in XIAP levels was observed. Thus, a caspase-dependent apoptotic cell death can be inferred. It exerted moderate effect on normal cells, HaCat, HBL-100 and NIH/3T3. In addition, at a sub-lethal dose, a marked reduction in chemoinvasive and migratory potential and prominently diminished angiogenesis both *in vitro* and *in vivo* was observed. On detailed analysis of the cellular pathways, we understood that EP4 blockade by GW627368X reduced PKA phosphorylation, which in turn lead to decreased CREB activation. This decreased PKA phosphorylation is directly responsible for enhanced Bax activity and in part reduces GSK3*β* phosphorylation. A decreased CREB transcriptional activity restricts the aberrant transcription of key genes like EP4, COX-2 and VEGF which ultimately control cell survival, proliferation and angiogenesis.

Owing to the interactive signaling between EP4 and EGFR, the phosphorylation status of downstream effectors like Akt, MAPK and GSK3*β* are also affected, which play important role in survival and proliferation (Supplementary Figure S1). We also confirmed the transactivation of EGFR by EP4 and the effect of GW627368X there in by exogenous overexpression of PGE2. PGE2 stimulated EP4 receptor which in turn significantly increased phosphorylation level of EGFR. Treatment with GW627368X, however, lowered EGFR phosphorylation in all cell lines. Inhibition of EGFR phosphorylation by GW627368X in turn reduced the phosphorylation level of Akt, MAPK and GSK3*β* significantly. *β*-catenin is an important transcription factor whose activity is controlled by Akt and GSK3*β.*^20^ On treatment with a sublethal dose, GW627368X was found to reduce the nuclear translocation of *β*-catenin in a time-dependent manner. In addition, we also observed a time-dependent decrease in cytoplasmic as well as cellular *β*-catenin. TCF-*β*-catenin activation is known to be associated with activation of variety of genes-like c-myc and cyclin D1.^20^ EGFR activation leads to an increased activator protein-1-mediated induction of COX-2 transcription *via* MAPK and enhanced synthesis of PGE2.^12^ Reduced 15-hydroxyprostaglandin dehydrogenase expression restricts PGE2 catabolism resulting in enhanced accumulation of PGE2 in the tumor vicinity.^11^ This explains the reduced PGE2 and VEGF secretion we observed in respective immunoassays and can be implicated in hindered angiogenesis.

As discussed earlier, EP4 transactivates and interacts with EGFR *via* multiple context specific pathways. Being a competitive EP4 antagonist, GW627368X prevents ligand receptor interaction when ambient PGE2 level is increased by exogenous addition. This in turn restricts EGFR transactivation and corresponding downstream pathways which explains the downregulation of pMAPK, pAkt and pGSK3*β* along with pEGFR. Disruption of cross talk between receptors is also validated by a similar downregulation of the effectors on blocking EGFR using mAb with or without the drug. Disruption of this cooperative signaling leads to reduced activity of transcription factors CREB and *β*-catenin resulting in reduced synthesis of survival factors. In xenograft model, a dose-dependent decrease in proliferating cancer was evident from dose-dependent reduction in tumor mass and volume and a marked decrease in Ki67 expression. A diminished CD31 specific staining signified decreased tumor angiogenesis. Reduced expression of pAkt, pMAPK, pEGFR and COX-2 confirmed our *in vitro* results.

It can therefore be inferred from our *in vitro* and *in vivo* results that GW627368X effectively diminishes tumor survival, motility, proliferation and angiogenesis by blocking EP4/EGFR interactive signaling in cervical cancer. Our results demonstrate EP4 as a potent therapeutic target in cervical cancer and can be effective in combination with conventional therapies to attain superior outcomes and to overcome complications associated with organ toxicities, therapeutic resistance and disease relapse.

## Materials and Methods

### Cell lines

Cervical cancer cells HeLa, SiHa and ME 180 were procured from National Centre for Cell Science (Pune, India) and maintained in 5% CO_2_ atmosphere, at 37 °C and 95% humidity in DMEM (Gibco-BRL, Rockville, MD, USA; HeLa and SiHa) and McCoy-5A (Sigma Aldrich, St. Louis, MO, USA; ME 180) supplemented with 10% heat inactivated FBS (Gibco-BRL)

### Reagents

GW627368X (Cayman Chemical Item Number 10009162, CAS 439288-66-1) and CJ-023423 (Cayman Chemical Item number 10010355, CAS 415903-37-6) were procured from Cayman chemicals, Ann Arbor, MI, USA, made into stock solutions of 10 mM in DMSO and stored at −20 °C, diluted into fresh medium before use. For western blot and IHC rabbit monoclonal anti-COX-2, anti-apoptosis-inducing factor, anti-BAX, anti-Bcl-2, anti-vascular endothelial growth factor receptor, anti-p-vascular endothelial growth factor receptor, anti-EGFR, anti-p-EGFR, anti-Akt, anti-p-Akt, anti-MAPK, anti-p-MAPK, anti-Ki67, anti-Ras, anti-p-Ras, anti-Raf, anti-p-Raf, anti-GSK3*β*, anti-p-GSK3*β*, anti-*β*Catenin, anti-p-*β*Catenin, anti-CD31(Cell Signaling Technology, Beverly, MA, USA), mouse monoclonal anti-*β*-actin (Cell Signaling Technology) and goat monoclonal anti-EP4 (Imgenex, Bhuvaneswar, India), horseradish peroxidase conjugated goat anti-rabbit immunoglobulin G and goat anti-mouse immunoglobulin G (Santa Cruz Biotechnology, Santa Cruz, CA, USA), Chemiluminescent peroxidase substrate, propidium iodide and MTT (Sigma Aldrich) ApopTag *In Situ* Apoptosis detection kit (Promega, Madison, WI, USA), Rhodamine phallaoidin (Invitrogen Corporation, CA, USA) Fetal bovine serum (FBS) (Gibco-BRL, Invitrogen Corporation, CA, USA), PGE2 immunoassay kit (Imgenex, India), Human VEGF quantikine ELISA kit (R&D systems, Minneapolis, MN, USA), and IHC detection system (Biogenex, Fremont, CA, USA) were purchased from corresponding companies. Stock solutions of propidium iodide and MTT were prepared by dissolving 1 mg of each compound in 1 ml phosphate-buffered saline. The solutions were stored in dark at 4 °C and used within 1 month. Stock concentrations of 10 mg/ml RNase A (Sigma Aldrich) was prepared and kept at −20 °C.

### Cell viability assay

Cell proliferation was determined by traditional MTT assay with slight modification and LIVE/DEAD assay.^21^ Cell viability was measured by MTT dye reduction assay at 24 and 48 h after GW627368X treatment(0-100 *μ*M) at 540 nm.^22^ The time dependent dose-response curves were analyzed using Prism software (GraphPad Prism, CA, USA). LIVE/DEAD assay was performed in drug treated cells (9,10 and 10 *μ*M for HeLa, SiHa and ME 180 respectively) for 12, 24 and 48 h using LIVE/DEAD Viability/Cytotoxicity kit (Life Technologies, Carlsbad, CA, USA) as per the manufacturer's instructions.^21^ The percentages of live and dead cells were calculated and plotted using Prism software (GraphPad Prism, CA, USA).

### Cell cycle analysis

Cells grown to 70-80% confluence were treated with GW627368X (9, 10, 10 *μ*M for HeLa, SiHa and ME 180 respectively) for varying time points, 70% ethanol fixed, RNaseA (100 *μ*g/ml) and propidium iodide at a final concentration of 40 *μ*g/ml added and incubated for 45 min at 37 °C. Cells were analyzed with FACS Vantage SE (BD Corporation, Franklin Lakes, NJ, USA). Results obtained were analyzed with Cell Quest software version 2.0 (BD).

### TUNEL assay

Occurrence of apoptosis was investigated by TUNEL assay on cells treated with 9, 10, 10 *μ*M GW627368X for HeLa, SiHa and ME 180 respectively for 0, 24 and 48 h and on tissue sections of excised tumors using ApopTag Peroxidase *in situ* Apoptosis Detection Kit (Promega) following kit protocol.^23^ Cells were analyzed by confocal laser scanning microscope at × 20 magnification.

### Morphological studies

Adherent monolayer cells were washed with phosphate-buffered saline, fixed with 4% paraformaldehyde and permeabilized with 0.1% triton X-100 for 1 min. Rhodamine phalloidin staining was performed to visualize cell cytoskeleton while DAPI was used to stain nuclei.^23^ Fluorescent images were captured under a Zeiss Observer Z1 microscope (Carl Zeiss, Germany) at × 20 magnification.

### Wound-healing assay

Monolayers of HeLa, SiHa and ME 180 were wounded using a pipette tip, cultured in presence or absence of GW627368X (IC_50_/3) for different time points till 48 h and cells invading the wound line were observed under an inverted phase-contrast microscope using × 10, Leica DMR, Germany. Wounds were quantified from distance between edges using Leica QWin software, Richmond, IL, USA.^22^

### Boyden chamber invasion assay

Filters (8 *μ*m) coated with Matrigel (20 *μ*g/ filter) were placed in Boyden chambers. ME 180 cells (1 × 10^5^), suspended in DMEM containing 0.1% BSA and treated with GW627368X, were placed on the top chamber. Conditioned medium from NIH/3T3 mouse fibroblast cells was used as chemoattractant source and placed in the bottom compartment. Following 24 h incubation at 37 °C, non-invading cells were scraped off, and cells migrated to lower surface of filter inserts were fixed using 100% methanol for 10 min and stained with haematoxylin.^21^ Results are expressed as percent of migrated cells as compared with control.

### Chick chorioallantoic membrane assay

Fertilized eggs were incubated at 37 °C in 60–70% relative humidity for 7 days after which 1–2 cm^2^ windows were opened and sterile round filter paper disks (5 mm in diameter, Whatman qualitative filter papers, Sigma-Aldrich, St. Louis, MO, USA) containing serum-free medium alone as control, VEGF (25 ng/ml), GW627368X or both (one third of IC_50_) was applied onto chorioallantoic membrane of individual embryos. After 2 days incubation, upper eggshell was removed and capillaries within 2.5 mm around the filter paper were observed and photographed under a stereomicroscope^22^ (Olympus, SZX16, Center Valley, PA, USA).

### HUVEC assay

Growth factor-depleted matrigel was applied to a 96-well tissue culture plate (50 *μ*l per well). After polymerization(37 °C, 1 h), HUVECs, serum starved for 2 h were harvested, washed with assay medium and 7.5 × 10^3^ cells/well (final volume, 50 *μ*l) were seeded on polymerized matrigel without or with drug(IC_50_/3), VEGF(25ng/ml) or both. The plate was incubated at 37 °C for 24 h, medium was aspirated and cells fixed in neutral buffered 10% formalin.^21^ Representative pictures were taken at × 10 magnification.

### Protein isolation and western blotting

Cervical cancer cells HeLa, SiHa and ME 180 were grown and treated with 9, 10 and 10 *μ*M GW627368X, respectively, for 12, 24 and 48 h or with 0.1% DMSO in cell culture dishes for 48 h. For phosphorylation studies, experimental plates were treated with same dose of GW627368X, whereas control plates were treated with 0.1% DMSO for 1 h. The cells were then stimulated with recombinant human EGF (25 ng/ml) for 30 min. The cells were scraped and lysed in Nonidet P-40 lysis buffer. Cell lysate containing 50 *μ*g protein was separated on sodium dodecyl sulfate–polyacrylamide electrophoretic gel, transferred to nitrocellulose membranes followed by blocking in 3% BSA for 2 h. The membranes were then incubated with primary antibodies overnight at 4 °C and then with horseradish peroxidase-conjugated secondary antibody for 2 h at room temperature.^22^ Proteins were visualized using chemiluminescence substrate and exposed to Kodak X-OMAT AR autoradiography film (Eastman Kodak, Rochester, NY, USA).

To validate the signaling mechanism involved, phosphorylation status of different protein molecules were studied after either stimulating or blocking the key receptors. In brief, overnight serum starved cells were stimulated with 100 nM PGE2 exogenously. Prostanoid receptor EP4 was blocked using GW627368X whereas EGFR was blocked using 20 ng/ml EGFR monoclonal blocking antibody. Relative activity of downstream regulators was studied by western blot after blocking or stimulating either of the receptors or in combination.

### Sub-cellular fractionation

Sub-cellular fractionation was performed as earlier reported with minor modification and western blotting was performed.^24^ Cells were washed with cold phosphate-buffered saline and pellet collected by centrifugation at 3000 r.p.m. for 10 min. Cells were incubated on ice for 15 min in hypotonic buffer A and homogenized. Nuclei were separated from the cytosolic fraction by centrifugation at 5000  r.p.m. for 5 min at 4 ºC. Cytosolic fraction was centrifuged at 40 000 r.p.m. for 30 min to pellet the crude membranes. Nuclei were washed and resuspended in nuclear protein extraction buffer,^24^ lysed with vigorous shaking for 20 min and centrifuged at 15 000 r.p.m. for 30 min to collect the soluble nuclear fraction.

### PGE2 and VEGF quantification by ELISA

For quantification of PGE2, cells were allowed to grow for 24 h and treated with 9, 10 and 10 *μ*M GW627368X for HeLa, SiHa and ME 180, respectively, in serum-free medium for 1 h followed by stimulation of the cells with 10 *μ*M arachidonic acid. After an incubation period of 24 h, PGE2 concentration in conditioned media was quantified using IMGENEX PGE2 EIA kit as per manufacturer's protocol. For VEGF quantification, cells were grown for 24 h and treated with same drug dosage or 0.01% DMSO and incubated for 24 h. To maintain stability of VEGF, treatment was carried out in medium supplemented with 2% FBS. VEGF concentration in conditioned medium was then measured using VEGF quantikine ELISA kit, R&D according to kit protocol.^25^

### Tumor xenograft

Female athymic nude mice (N:NIH(S)) purchased from National Institute of Nutrition, Hyderabad, India were housed in National Institute of Cholera and Enteric Diseases, Kolkata, India in accordance with institutional guidelines. Experiments were approved by Institutional Animal Ethical Committee and were conducted observing all Animal Ethics Regulation streamlined by the Institutional Animal Ethics Committee concerned under guidance of the Committee for the purpose of Control and Supervision of Experiments on Animals, Govt. of India. Animals were allowed free access to standard laboratory rodent food and water under good laboratory conditions (temperature 25±2 °C; relative humidity 50±20%) with dark and light cycle (12/12 h). Mice (6–8-week-old) having body weight around 20–22 g were used for experiment. Exponentially growing ME 180/SiHa cells were harvested and a tumorigenic dose of 2.5 × 10^7^ cells in matrigel 0.5 mg/ml was injected subcutaneously in flanks of the mice.^23^ After ME 180 tumors attained a volume of about 100mm^3^, mice were randomized into five groups of five mice per group. In case of SiHa xenograft, as there was very poor tumor formation, an initial volume of only about 10 mm^3^ could be attained. Groups of animals were treated with varying doses of drugs (i) control; (ii) 2 mg/kg; (iii) 4 mg/kg; (iv) 6 mg/kg; and (v) 10 mg/kg. Treatment was administered for a period of 4 weeks (3 times a week) using an oral gavage. Tumor volume was measured twice a week by a side callipers, using equation: *V*=(*A*) (*B*^2^)π /6, where *A* was the length of longest aspect of tumor, and *B* was the length of tumor perpendicular to *A*.^21^

### Immunohistochemistry

Tumor specimen excised from respective groups of ME 180 xenografts were fixed in 10% formalin, embedded in paraffin and sectioned. For immunohistochemical analysis, tissue sections were deparafinized followed by antigen retrieval. IHC for specific proteins were carried out on tumor tissue sections using Biogenex IHC detection system according to manufacturer's guidelines with different antibodies including anti–pAkt, anti–Akt, anti–Ki67, anti–CD31, anti–pEGFR, anti-EGFR, anti-pMAPK, anti-MAPK and anti-COX-2 proteins.

## Abbreviations

COX: cyclooxygenase
VEGF: vascular endothelial growth factor
PGE2: prostaglandin E2
PKA: protein kinase A
CREB: cAMP response element-binding protein
GSK3: glycogen synthase kinase 3
TUNEL: terminal deoxynucleotidyl transferase dUTP nick-end labeling
EGFR: epithelial growth factor receptor
MAPK: mitogen-activated protein kinases
ELISA: enzyme linked immunosorbent assay
IHC: immunohistochemistry
MTT: 3-(4,5-dimethylthiazol-2-yl)-2,5 diphenyltetrazolium bromide
CJ: CJ-023423
