# Supplementary material for: GW627368X inhibits proliferation and induces apoptosis in cervical cancer by interfering with EP4/EGFR interactive signaling
Source: Cell Death Dis. 2016 Mar 24;7(3):e2154–. doi: 10.1038/cddis.2016.61 (PMC4823960; doi:10.1038/cddis.2016.61)
Supplement: Supplementary Figure 1 [file cddis201661x1.doc]

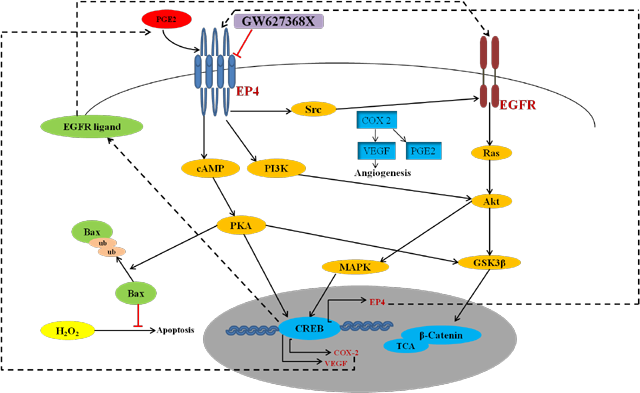


**Supplementary figure 1. Schematic representation of EP4/EGFR interactive signaling and mechanism of action of GW627368X. Being a competitive EP4 receptor antagonist, GW627368X prevents ligand receptor binding between PGE2 and EP4 thereby inhibiting downstream PKA/CREB pathway. Blockade of EP4 also inhibits EGFR transactivation and consequent downstream pathways thereby lowering CREB and β-catenin transcriptional activity reducing synthesis of survival factors, hence hindering proliferation**
